# Axons in the Chick Embryo Follow Soft Pathways Through Developing Somite Segments

**DOI:** 10.3389/fcell.2022.917589

**Published:** 2022-07-08

**Authors:** Julia Schaeffer, Isabell P. Weber, Amelia J. Thompson, Roger J. Keynes, Kristian Franze

**Affiliations:** ^1^ Department of Physiology, Development and Neuroscience, University of Cambridge, Cambridge, United Kingdom; ^2^ Inserm, U1216, Grenoble Institut Neurosciences, Univ. Grenoble Alpes, Grenoble, France; ^3^ Institute of Medical Physics, Friedrich-Alexander-Universität Erlangen-Nürnberg, Erlangen, Germany; ^4^ Max-Planck-Zentrum für Physik und Medizin, Erlangen, Germany

**Keywords:** AFM, axon pathfinding, tissue stiffness, somite polarity, nervous system development, spinal motor axons, stiffness patterns

## Abstract

During patterning of the peripheral nervous system, motor axons grow sequentially out of the neural tube in a segmented fashion to ensure functional integration of the motor roots between the surrounding cartilage and bones of the developing vertebrae. This segmented outgrowth is regulated by the intrinsic properties of each segment (somite) adjacent to the neural tube, and in particular by chemical repulsive guidance cues expressed in the posterior half. Yet, knockout models for such repulsive cues still display initial segmentation of outgrowing motor axons, suggesting the existence of additional, yet unknown regulatory mechanisms of axon growth segmentation. As neuronal growth is not only regulated by chemical but also by mechanical signals, we here characterized the mechanical environment of outgrowing motor axons. Using atomic force microscopy-based indentation measurements on chick embryo somite strips, we identified stiffness gradients in each segment, which precedes motor axon growth. Axon growth was restricted to the anterior, softer tissue, which showed lower cell body densities than the repulsive stiffer posterior parts at later stages. As tissue stiffness is known to regulate axon growth during development, our results suggest that motor axons also respond to periodic stiffness gradients imposed by the intrinsic mechanical properties of somites.

## Introduction

**FIGURE 1:**
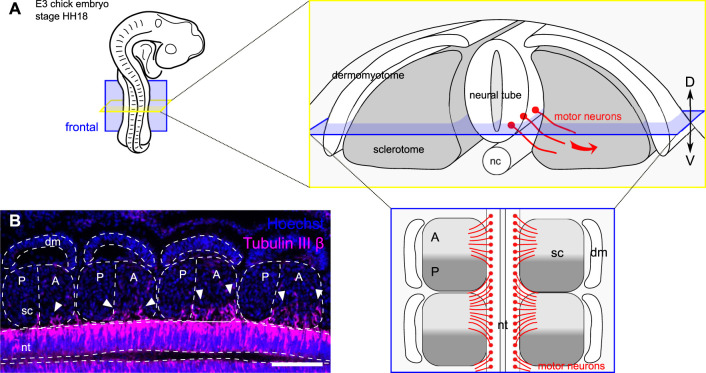
Motor axons grow out of the neural tube in a segmented fashion. **(A)** Schematic representation of the developing peripheral nervous system in chick embryo embryonic day 3 (E3), stage HH18. Motor neurons project their axon out of the neural tube, in the sclerotome, along a ventral-lateral trajectory. The frontal plane (in blue) is used to study segmentation of outgrowing motor axons. (D) dorsal; V: ventral. **(B)** Immunohistochemistry on a frontal section of a chick embryo showing motor axons (Tubulin III β, red) growing out of the neural tube (nt) and cell nuclei (Hoechst, blue) in somite segments. Scale bar = 100 μm dm: dermomyotome; sc: sclerotome; nc: notochord.

The segmentation process underlying vertebral column development begins early during embryogenesis, through the formation of the mesodermal somites. Somites are clusters of cells bilaterally paired that arise transiently from the mesenchymal pre-somitic mesoderm (PSM) on each side of the neural tube along the antero-posterior axis (Saga and Takeda, 2001). The newly formed somites consist of columnar epithelial cells arranged in a sphere with a mesenchymal core. In response to signals from the notochord and the floor plate of the neural tube, somites undergo a partial epithelial-to-mesenchymal transition (EMT) to differentiate into the mesenchymal sclerotome (ventral) and the epithelial dermomyotome (dorsal) (Figure 1A). This differentiation happens sequentially from the anterior to the posterior end of the body, similar to somitogenesis. The dermomyotome will give rise to dermis of the back and skeletal muscles of the trunk, while the sclerotome will give rise to cartilage and bone of the vertebrae and ribs (Christ et al., 2004; Scaal, 2016).

In addition to the dorso-ventral regionalisation, each sclerotome gets polarized along the anterior-posterior (or rostro-caudal) axis. This anterior-posterior division is already set in the anterior end of the pre-somitic (unsegmented) mesoderm (in the prospective segment) (Saga et al., 1997; Buchberger et al., 1998) and becomes morphologically visible as early as somite stage V and materialized by an intrasegmental boundary known as the von Ebner’s fissure. This early segmentation process allows correct patterning and integration of the developing peripheral nervous system (PNS) (i.e., afferent and efferent nerve fibers) within the vertebral column (Kuan et al., 2004). A readout of this phenomenon is the ventro-lateral outgrowth of motor axons from the neural tube, which grow together through the anterior halves of the sclerotomes while avoiding the posterior halves (Figure 1A). This binary system ensures that the peripheral nervous system develops without obstruction by the future cartilage and bones of the vertebral column.

The segmentation of motor neurons is known to be controlled by the intrinsic molecular polarization of each sclerotome into a growth-permissive region (anterior half) and a growth-repulsive region (posterior half) (Stern and Keynes, 1987; Kuan et al., 2004) (Figure 1B). For decades, researchers have focused on identifying key guidance molecules that ensure the correct segmentation of the developing peripheral nervous system. Notably, repellent cues are expressed by posterior half-sclerotome cells in order to exclude navigating axons from these “no-go” areas and restrict their growth to specific exit points of the future vertebral column. These include some canonical repulsive guidance cues identified in the posterior somite half, including Semaphorin 3A and Ephrin-B1, which contribute to the blockade of motor axon growth in that region (Kuan et al., 2004; Bonanomi and Pfaff, 2010). However, despite a strong repulsive activity of these molecules *in vitro*, their expression *in vivo* does not fully explain the initial segmentation of spinal nerve fibers (Koblar et al., 2000; Huber et al., 2005; Roffers-Agarwal and Gammill, 2009).

For example, knockout of certain repulsive axon guidance factors does not abolish the segmented pattern of spinal nerves without a complete “caudalisation” or “rostralisation” of the somites (Davy and Soriano, 2007; Lewcock et al., 2007; Bai et al., 2011). While Nrp1^Sema-^ knockin (where specific binding of Semaphorin 3A to its receptor Neuropilin-1 is selectively abolished) and Nrp2-knockout mice exhibit large defasciculation in spinal motor projections, and these defects are enhanced in Nrp1^Sema-^;Nrp2^−/−^ double mutants, the initial segmentation of motor axons leaving the neural tube is still preserved (Huber et al., 2005). This suggests the existence of additional mechanisms regulating the initial segmented pattern of motor axons as they grow out of the neural tube and exit the vertebral column.

In order to grow through the tissue, axons have to exert forces on their environment and mechanically interact with it. Accordingly, local tissue stiffness contributes to controlling axon growth *in vivo*. Xenopus retinal ganglion cell axons, for example, grow along a stiffness gradient through the developing brain, avoiding stiffer areas. Perturbations of mechanosensitive ion channels or tissue stiffness leads to aberrant axon growth patterns with severe guidance defects (Koser et al., 2016; Thompson et al., 2019). To test if in the somite system each half-sclerotome may exhibit distinct mechanical properties contributing to the preference of motor axons to grow through the anterior half-sclerotome, we here recorded stiffness maps of dissected chick somite strips using atomic force microscopy (AFM)-based indentation measurements. We found that posterior half-sclerotomes, which axons avoid, are stiffer than anterior half-sclerotomes, and that this difference in local tissue stiffness arises prior to initial outgrowth of motor axons and prior to the onset of differences in local cell densities between the two regions, suggesting a role for somite mechanics in regulating neuronal fasciculation and growth.

## Materials and Methods

### Embryo Manipulation and Dissection

Wild-type chick eggs were obtained from Winter Egg Farm. Eggs were incubated at 37.5 °C until the embryos reached the required stage. The development of embryos was assessed according to Hamburger Hamilton (HH) stages (Hamburger and Hamilton, 1992). The somite stage was assessed according to (Christ and Ordahl, 1995), with roman numerals giving an index of the *n*th most recently formed somite (from posterior to anterior).

### Atomic Force Microscopy Indentation Measurements

**FIGURE 2:**
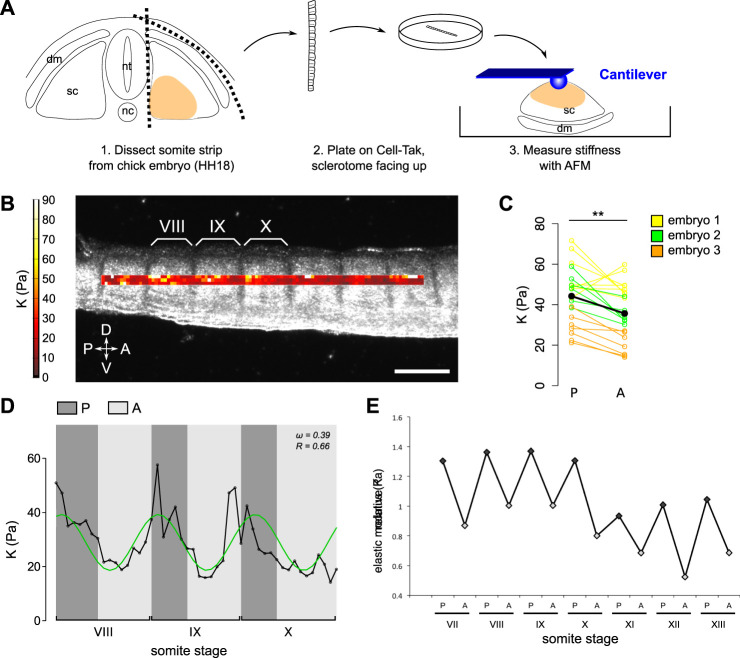
Posterior half-sclerotomes are stiffer than anterior half-sclerotomes. **(A)** Schematic representation of workflow to measure sclerotome stiffness with AFM. dm: dermomyotome; sc: sclerotome; nt: neural tube; nc: notochord. The colored region is the region of interest for AFM-based measurements. **(B)** Image of chick embryo with overlaid AFM-based stiffness map of sclerotome tissue along the antero-posterior axis. Color encodes the apparent reduced elastic modulus K assessed at an indentation force of 3 nN. Somite stage is indicated in roman numerals. Scale bar: 200 μm. Sclerotome tissue displayed a periodic stiffness gradient corresponding to the spatial periodicity of anterior and posterior halves. (D) dorsal; V: ventral; P: psoterior; (A) anterior. **(C)** Mean K in anterior half-sclerotomes (A) and in corresponding posterior half-sclerotomes (P) in 21 sclerotomes of three embryos. The black dots represent the mean of all values. Sclerotomes were significantly stiffer in the posterior half compared to their anterior half, along which axons grow (paired *t*-test, **p = 0.0012). **(D)** Stiffness distribution for three consecutive segments (somite VIII, IX, and X). The black line represents mean K at each 15 μm step along the antero-posterior axis, the green line represents the sinusoidal regression of the data. The plot is representative of N = 5 embryos. **(E)** Example of relative elastic modulus *K* values along the antero-posterior axis as a function of somite stage, showing that posterior halves are stiffer than anterior halves at least from somite stage VII.

Somite strips were microdissected from chick embryos, embryonic day 3 (HH18), and plated on Cell-Tak Cell Tissue Adhesive (Corning^®^, Sigma) in a plastic TPP Petri dish (Sigma- Aldrich), with sclerotomes facing up (Figure 2A). AFM indentation measurements were performed with a JPK Cellhesion 200 (JPK Instruments AG, Berlin, Germany) placed on an inverted optical microscope (Axio Observer. A1, Carl Zeiss Ltd., Cambridge, United Kingdom) and a motorized xy stage. Tissue was observed through an upright microscope (Axio Zoom. V16, Carl Zeiss Ltd., Cambridge, United Kingdom) mounted above the AFM. Tipless silicon cantilevers (Arrow-TL1; NanoWorld, Switzerland, spring constant ∼0.03–0.04 N m^−1^) were used, and 37 μm diameter polystyrene beads (microParticles GmbH, Germany) were glued to the cantilevers as probes. Force–distance curves were taken with an approach speed of 10 μm/s and a set force of 3 nN in an automated raster scan using the motorized stage with 15 μm step size. Using the method described in (Gautier et al., 2015), the contact point was found and subsequently the indentation depth *δ* was calculated by subtracting the cantilever deflection d from the piezo translation z after contact *δ* = z−d. The apparent reduced elastic moduli *K = E*/(1−*v* ^2^), where *E* is the Young’s modulus and *v* is the Poisson’s ratio, were extracted from the force–distance curves by fitting the contact portion of curves to a Hertz contact model between a sphere and an infinite half space using a customized MATLAB (Mathworks) routine previously described (Christ et al., 2010).

### Immunohistochemistry

After fixation and dissection of the vitelline membrane and the amnion, embryos were washed in PBS, then dehydrated in ethanol and incubated in histosol (National Diagnostics). Embryos were immersed in paraffin wax (Raymond Lamb) at 65°C. Embryos were positioned in plastic Peel-A-Way molds (Sigma) for embedding, then sectioned with a rotary microtome (Leica). 6 μm sections were mounted on Superfrost Plus glass slides (VWR) on water, and dried overnight. For histology, sections were immersed successively in histosol and ethanol, then rehydrated in PBS. Sections were blocked with PBS 5% goat serum for 1 h, then incubated with anti-Tubulin 3β antibody (TUJ1) (BioLegend, cat. n. 801202) diltued 1:250 in blocking buffer overnight at 4°C. Sections were incubated with Alexa-conjugated secondary antibody 2 h at room temperature. Nuclear staining was performed with Hoechst (Roche) diluted 1:5000 in PBS. Slides were mounted with Fluoromount-G (SouthernBiotech). Sections were observed with fluorescence microscopy (Zeiss Axioplan 2), and images taken with Q-Capture Pro 6.0. Cell density was determined by counting the number of nuclei in each region of interest (anterior and posterior halves of each sclerotome). For each segment, a region of interest (ROI) of fixed area was defined within the half-sclerotome (anterior and posterior), which were easily distinguishable based on the characteristic segmentation of the dermomyotome that outlines individual segments, and the number of nuclei was manually counted. The number of nuclei was normalized to the average number of nuclei in all ROIs.

### Data Analysis and Representation

Data analysis and representation were performed with R (R Core Team, 2014). For plot representation of relative elastic moduli *K* as a function of somite stage, the median value of all values measured within one half-segment was calculated. For the periodic representation of *K*, we took the mean of three values at each step along the A/P axis. The sinusoidal fit of the data was performed using the *nls* function of the R package *stats*, which estimates nonlinear least-squares parameters of a nonlinear model. The function passed to the sinusoidal regression model is: *y(t)* = *C* + α*sin(*ωt* + *φ*)*.* Starting parameters were defined as: *C*_*0*_ = (max*(y) +* min*(y)*)/2; *α*_*0*_ = max*(y)*—*C*_*0*_; *ω*_*0*_ *=* 2*π/15, as the length of one segment corresponds to 15 points of measurements on average; *φ*_*0*_ = {*0*; *π*}, with *0* if the dataset starts with a posterior half-sclerotome, and *π* if it starts with an anterior half-sclerotome. Correlation was calculated with the function *fitted* and the function *cor* of the R package *stats*. A total of five embryos (one somite strip per embryo) were analysed. For plot representation of relative cell density, data are represented as average ± standard error of the mean.

### Statistical Analysis

For comparison of anterior and posterior half-sclerotomes, power calculations and statistical analysis were performed with R (R Core Team, 2014). For each condition (anterior or posterior), the Shapiro-Wilk test for normality was used to assess normal distribution of data (*p*-value ≥ 0.01). *p*-values were calculated with two-tailed *t*-test to compare the two regions. Datasets that were not normally distributed were subjected to a log10 transformation or compared with a Wilcoxon rank sum test.

## Results

In order to investigate mechanical properties of sclerotomes at cellular resolution, elasticity maps were taken on dissected chick somite strips using AFM-based indentation measurements. Somite strips were separated from the neural tube in chick embryos at embryonic day 3 (HH18) and plated on the dish with the ventro-medial surface facing up, giving access to the sclerotomal region (Figures 2A,B). Tissue stiffness was quantified as the apparent reduced elastic modulus *K*, where a larger *K* value indicates stiffer tissue. Elasticity maps were superimposed on bright field images of the tissue, highlighting morphological subdivisions (Figure 2B). The analysis of the distribution of apparent elastic moduli *K* revealed that posterior half-sclerotomes were on average ∼30% stiffer than corresponding anterior half-sclerotomes (mean values: 36 Pa versus 44 Pa; paired *t*-test, *p* = 0.0012) (Figure 2C). Thus, motor axons preferentially grew through softer tissue.

The spatial mapping of the apparent elastic moduli revealed periodic patterns of higher and lower tissue stiffness along the antero-posterior (A/P) axis. To visualize this periodicity, the mean of three values along the dorso-ventral (D/V) axis was plotted as a function of distance along the A/P axis (Figure 2D). We focused on the 8th, 9th, and 10th most recent somites [somites VIII, IX, and X according to Christ and Ordahl’s staging system (Christ and Ordahl, 1995)], at the time just before the initial outgrowth of motor axons from the neural tube (somite X, XI). We performed a non-linear regression analysis to fit the datasets to a sinusoidal function. The sinusoidal regression supported the observation that the spatial periodicity in low and high stiffness corresponded to the length of one somite segment. To determine the parameters of the sinusoidal fit, a theoretical value of the periodicity *ω* was passed to the sinusoidal fit function, as follows: *ω*_*0*_ is 2*π/15 ≈ 0.42—because the length of one segment corresponds to 15 points of measurements on average. Based on the sinusoidal regression, the estimation for the experimental periodicity *ω* was 0.45 ± 0.064 with Pearson’s correlation coefficient R = 0.59 ± 0.085 (N = 5 embryos) (Figure 2D). This experimental value confirmed the periodic nature of the stiffness intrinsic to each somite segment. Moreover, our measurements revealed that the higher stiffness in posterior half-segments occurred as early as somite VII (the seventh most recently formed somite) (Figure 2E), which precedes motor axon initial outgrowth that is observed not earlier than somite X, XI. Together, these data revealed a new parameter of intrinsic difference between the two half-sclerotomes in the developing chick embryo, which could contribute to the segmented patterning of outgrowing motor axons.

**FIGURE 3:**
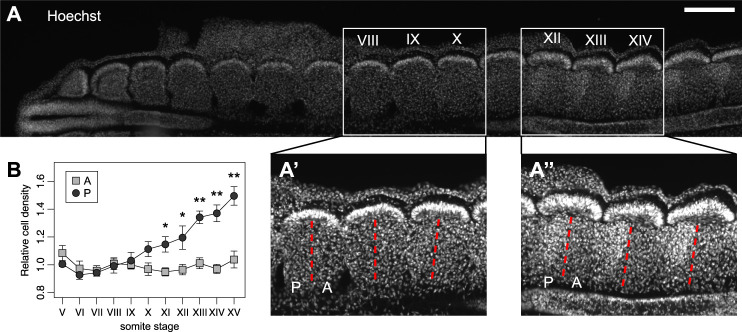
Intrasegmental differences in tissue stiffness arise before motor axon outgrowth and before differential cell density. **(A)** Immunohistochemistry on a frontal section of a chick embryo showing the gradually higher density of cell nuclei (Hoechst, blue) along the antero-posterior axis. Scale bar: 200 μm. (A′) and (A″) zoom of somite stages VIII-X and XII-XIV, respectively. **(B)** Quantification of cell densities in anterior and posterior halves of somite stages V–XV. Each point represents the average of cell densities relative to the anterior halves for each embryo. Error bars represent standard error of the mean. Wilcoxon rank sum test, *p < 0.05, **p < 0.01. N = 5 embryos.

The intrasegmental difference of stiffness may be due to differences in the composition of the extracellular environment (Swift et al., 2013; Moeendarbary et al., 2017) and/or in differences in cell density (Koser et al., 2015; Thompson et al., 2019). Indeed, we quantified the number of nuclei in each sclerotome half and we found that cell density increased sequentially in the posterior half starting at somite stage X (10th most recently formed somite) (Figures 3A,B). However, the increase in stiffness of the posterior half-sclerotome preceded its higher cell density (Figures 2E–3B), suggesting that other factors contribute to the differential tissue mechanics. Altogether, our results reveal a spatial correlation between differential tissue stiffness inside each sclerotome and the preferential path of motor axons through the anterior, softer half-sclerotome. Temporally, the difference in stiffness arises before the initiation of motor axon outgrowth, providing an additional potential layer of regulation of axon guidance in the PNS segmentation process.

## Discussion

In the past three decades, much effort has been made to determine the identity of repulsive chemical guidance cues expressed at the surface of posterior half-sclerotome cells (Kuan et al., 2004; Bonanomi and Pfaff, 2010). Several canonical guidance molecules participate in the segmentation of motor axon outgrowth, while not fully accounting per se for the segmentation process in the developing spinal cord (Koblar et al., 2000; Huber et al., 2005; Roffers-Agarwal and Gammill, 2009). Recently, the identity of a peanut agglutinin-binding protein has been unraveled as cell surface protein disulfide isomerase (PDI), which mediates the contact-repulsion mechanism of posterior half-sclerotomes (Cook et al., 2020). However, alongside chemical signalling, mechanical signals may also participate in the joint ingrowth of spinal axons into the anterior half-segment while avoiding the posterior segment.

**FIGURE 4:**
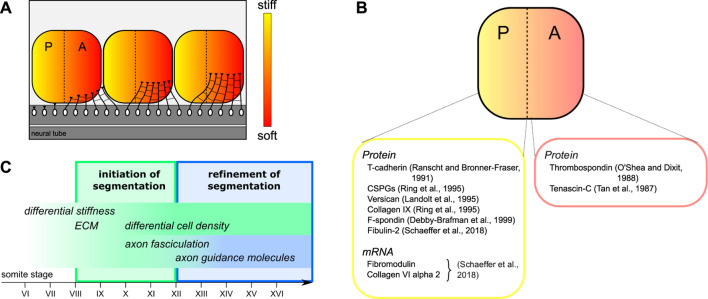
Graphical summary of intrasegmental stiffness polarity during segmentation of motor axon outgrowth. **(A)** Schematic representation of motor axons growing out of the neural tube. Our results suggest that axons respond to differential stiffness and turn into the softer anterior half-sclerotome before fasciculating to form nerve bundles. **(B)** Examples of ECM and ECM-associated components showing a polar distribution in the sclerotome during development. References are given for stainings by immunolabelling (Protein) and by *in situ* hybridization (mRNA). **(C)** Proposed model of initial segmentation of motor axons controlled by the periodic gradient of stiffness along the antero-posterior axis from somite stage VIII, preceding the initial motor axon outgrowth.

In our study, we found that the sclerotome is intrinsically mechanically heterogeneous, with its posterior half being significantly stiffer than its axon growth-promoting anterior half. The differential stiffness within each sclerotome segment arises before the onset of antero-posterior-segmented motor neuron outgrowth from the neural tube. Hence, growing axons are exposed to different mechanical signals. These results provide an additional layer of complexity in the regulation of guidance and connectivity of growing axons. Together with differential gene expression of guidance and guidance-associated cues, these properties may contribute to the patterning of the developing nervous system. In the developing Xenopus brain, tissue stiffness contributes to controlling retinal ganglion cell (RGC) growth and pathfinding; RGC axons grow along a stiffness gradient and turn towards softer brain tissue *in vivo* (Koser et al., 2016; Thompson et al., 2019). Here, motor axons of the developing spinal cord turn into the anterior half-sclerotome, which provides a much softer environment if compared to the growth-inhibiting posterior half-sclerotome (Figure 4A). Hence, our results support the idea that motor axons respond to differential mechanical properties of the sclerotomal tissue to orient their trajectories during development of the peripheral nervous system. This segmented axon pattern is maintained during the maturation of the sclerotome and formation of the nerve motor and sensory roots, and eventually until cartilage condensation in the vertebrae at later stages of morphogenesis. Whether the stiffness polarity identify in this study is maintained at later stages of embryonic development remains to be determined.

The differential stiffness within each segment correlated with differential cell body densities in the tissue at later stages (Figure 3). In the vertebrate embryo, the morphology of the two sclerotome halves differs progressively from somite X. The morphological difference of the two poles is characterized by a higher cell density in the posterior half-sclerotome (Figure 3) (Mansouri et al., 2000), which becomes more condensed as the sclerotome matures. Indeed, cell proliferation is increased in the posterior half-sclerotome of chick and mammalian embryos, and the higher proliferation rate is maintained at later stages (Wilting et al., 1994). In fact, cells of the posterior half-sclerotome undergo progressive condensation under the action of the transcription factor Uncx4.1, which is restricted to the posterior half. Uncx4.1 is furthermore regulating differential adhesive properties of cells in the two halves of the polarized sclerotome (Stern and Keynes, 1987; Mansouri et al., 2000), overall leading to denser tissue in the posterior half-sclerotome, which might also contribute to limiting axon ingrowth.

Our finding that differential tissue stiffness is observed already from somite VII, i.e., before differences in anterior vs. posterior cell densities arise, suggests that the polarized sclerotome stiffness does not solely depend on local cell densities. The intrinsic mechanical polarity of the sclerotome may instead arise the differential expression of extracellular matrix (ECM) molecules (Swift et al., 2013; Moeendarbary et al., 2017; Segel et al., 2019). Consistent with this idea, several ECM molecules were identified as differentially expressed in the two half-sclerotomes in previous studies (Perris and Perissinotto, 2000; Kuan et al., 2004). In one study, peanut lectin (peanut agglutinin, PNA), which binds to extracellular glycoproteins with high collapse-inducing activity, was used as a preferential marker for the posterior half-sclerotome (Davies et al., 1990). Other posterior-restricted ECM components include chondroitin sulfate proteoglycans (Ring et al., 1995), versican (Landolt et al., 1995), collagen IX (Ring et al., 1995) and F-spondin (Debby-Brafman et al., 1999), while, conversely, tenascin-C (Tan et al., 1987) and thrombospondin (O’Shea and Dixit, 1988; Tucker et al., 1999) are preferentially expressed in the anterior half-sclerotome (Figure 4B). In addition, our recent transcriptomic profiling of anterior versus posterior half-sclerotomes revealed a strong enrichment of ECM proteins in the subset of genes differentially expressed between the two halves (Schaeffer et al., 2018). For example, we found a strong enrichment of laminin alpha 4 and collagen IX alpha 1 in the anterior half; while fibulin-2, fibromodulin and collagen VI alpha 2 are strongly enriched in the posterior half (Schaeffer et al., 2018) (Figure 4B). Therefore, ECM and ECM-associated proteins may contribute to the extracellular structure and organisation of the tissue, to the spatial distribution of chemical guidance cues, and to the intrinsic polarity of adhesive and mechanical properties within each segment.

When axons encounter stiffness gradients, they turn towards softer tissues (Koser et al., 2016; Thompson et al., 2019; Oliveri et al., 2021), similarly to the turning of motor axons towards softer half-sclerotomes observed in the present study. Neuronal mechanosensitivity is mediated, amongst others, by adhesion complexes coupling neurons to the ECM (Wang et al., 2021). Hence, neuronal adhesion molecules play a central role in axon growth. They enable force transmission to the environment required for axon growth through mechanical coupling (i.e., adhesion) of neurons to the ECM or other cells, and their activity may be regulated by both chemical and mechanical signals.

In sum, we show that the antero-posterior subdivision of somites is characterized by an intrinsic polarity in tissue stiffness, with anterior halves being softer than posterior halves. This binary pattern precedes motor axon initial outgrowth, as well as the progressive densification of cells in the posterior half-sclerotome (Figure 4C). As they grow out, motor axons find their way into the softer anterior half, which allows both axon growth and formation of spinal nerve bundles. Thus, this early differential stiffness pattern may considerably contribute to the initial segmentation of motor axon outgrowth.

## Data Availability

The raw data supporting the conclusion of this article will be made available by the authors, without undue reservation.
